# The Brazilian Transition in Differentiated Thyroid Carcinoma Management: A 25-Year Nationwide Analysis of Declining High-Activity Radioiodine Use (2000-2024)

**DOI:** 10.20945/2359-4292-2026-0038

**Published:** 2026-04-01

**Authors:** José Miguel Dora, Leonardo Barbi Walter, André B. Zanella, Iuri Goemann, Rafael Selbach Scheffel, Ana Luiza Maia

**Affiliations:** 1 Unidade de Tireoide, Hospital de Clínicas de Porto Alegre, Faculdade de Medicina, Universidade Federal do Rio Grande do Sul, Porto Alegre, RS, Brasil; 2 Divisão de Medicina Interna, Hospital de Clínicas de Porto Alegre, Porto Alegre, RS, Brasil; 3 Divisão de Endocrinologia, Hospital Nossa Senhora da Conceição, Grupo Hospitalar Conceição, Porto Alegre, RS, Brasil; 4 Departamento de Medicina Interna, Faculdade de Medicina, Pontifícia Universidade Católica, Porto Alegre, RS, Brasil; 5 Faculdade de Medicina, Universidade do Vale do Rio dos Sinos, São Leopoldo, RS, Brasil; 6 Departamento de Farmacologia, Instituto de Ciências Básicas da Saúde, Universidade Federal do Rio Grande do Sul, Porto Alegre, RS, Brasil

**Keywords:** Differentiated thyroid carcinoma, radioiodine, treatment

## Abstract

**Objective:**

To analyze temporal trends in radioiodine (RAI) prescription patterns for differentiated thyroid carcinoma (DTC) in Brazil from 2000 to 2024, assessing adherence to evolving clinical guidelines and identifying opportunities for practice optimization.

**Materials and methods:**

This retrospective study utilized data from the Brazilian Unified Health System (Datasus) to evaluate RAI prescriptions, categorized by activity: low (30 and 50 mCi), high (100 and 150 mCi), and very high (200 and 250 mCi). Population-adjusted rates, procedure-adjusted ratios (RAI/oncologic thyroidectomies and RAI/new cases), and activity-level trends were analyzed.

**Results:**

Three distinct phases emerged: ^(1)^ 2000-2007, marked by increasing very high-activity RAI use (≥ 200 mCi); ^(2)^ 2008-2015, peak utilization with initial diversification (introduction of 30/50 mCi in 2014); and ^(3)^ 2016-2024, significant de-escalation, with high/very high-activity prescriptions declining by 34.2% and low-activity use increasing by 163%. By 2024, RAI distribution comprised 15.3% very high-, 67.1% high-, and 17.6% low-activity prescriptions. The RAI/new cases ratio fell sharply from 0.63 (2010) to 0.25 (2024), and RAI/oncologic thyroidectomies dropped from 1.22 (2013) to 0.70 (2024), reflecting more selective prescription.

**Conclusion:**

Brazilian medical practice has increasingly aligned with international DTC guidelines, showing a decline in RAI use and a shift toward low-to-high RAI activities. This suggests broader adoption of risk-adapted strategies. Notwithstanding, high-dose RAI still predominates, pointing that dissemination of standardized treatment protocols could further enhance DTC care within the Brazilian health system.

## INTRODUCTION

Differentiated thyroid carcinoma (DTC), comprising papillary, follicular and oncocytic subtypes, accounts for most of thyroid malignancies and was responsible for almost one million new cancer diagnoses worldwide in 2025 ^(1)^. The cornerstone of treatment for DTC is a multimodal approach, fundamentally centered on surgical resection of the thyroid gland and, when appropriate, cervical lymph node dissection. Radioiodine therapy (RAI) may then be indicated for ablation of remaining tissue, as adjuvant therapy in intermediate/high-risk cases, or as treatment in persistent/recurrent disease ^(2)^.

Recent randomized controlled trials have provided high-level evidence supporting the safety of omitting RAI in patients with low-risk DTC. The IoN ^(3)^ and ESTIMABL2 ^(4)^ trials demonstrated the non-inferiority of surgery without RAI therapy compared with RAI administration regarding recurrence and disease-free survival. In parallel, the 2025 American Thyroid Association (ATA) guidelines highlighted the potential toxicities associated with high RAI activities-including salivary gland dysfunction, lacrimal damage, risk of infertility, and secondary malignancies-and recommend careful patient selection and dose optimization to minimize harm ^(5)^. Together, these data support the de-escalation of RAI therapy, emphasizing the importance of aligning clinical practice with contemporary evidence to optimize patient outcomes while reducing unnecessary interventions.

A previous study by Schwengber and cols. (2020) provided a comprehensive analysis of RAI utilization patterns in Brazil from 2000 to 2018, revealing a concerning variability in prescription practices and a gradual, yet insufficient, decline in very high/high-activity RAI use ^(6)^. Since then, the indications for RAI prescription based on the patient’s risk of recurrence have been emphasized. Nonetheless, emerging data on RAI-related cost-effectiveness have reinforced the need for its judicious use ^(7)^.

This study analyzes nationwide RAI prescription trends for DTC in Brazil from 2000 to 2024. By extending the timeline beyond our previous work, we seek to evaluate the current state of adherence to risk-adapted guidelines and identify persistent gaps between evidence and practice in the post-operative management of DTC.

## MATERIALS AND METHODS

This retrospective study examined patterns of RAI utilization for DTC management in Brazil from 2000 to 2024, utilizing data from the Brazilian Unified Health System (SUS). The primary data sources included the Department of Informatics of SUS (Datasus, http://datasus.saude.gov.br) from where we extracted annual counts of oncologic thyroidectomies and RAI prescriptions for DTC categorized by activity level: low (30 and 50 mCi), high (100 and 150 mCi), and very high (200 and 250 mCi), as detailed previously ^(6)^.

Population estimates for standardization were obtained from the Brazilian Institute of Geography and Statistics (IBGE, https://ibge.gov.br/), while annual projections of new DTC cases were derived from the Brazilian National Cancer Institute (INCA, https://www.inca.gov.br/) using established epidemiological methods.

Briefly, the methodology adopted by INCA for estimating cancer incidence in Brazil is primarily based on projecting data from high-quality Population-Based Cancer Registries (PBCRs) to cover the entire national territory. Since PBCRs do not cover all regions, statistical models, often using mean rates or regression techniques, are employed. These models correlate the available local incidence rates with sociodemographic and geographic indicators from uncapped areas. The projected incidence rates are then applied to the official population projections for the target year, generating the estimated number of new cases for each type of cancer nationwide. This approach enables a comprehensive and reasoned estimate, despite the absence of complete real-world data across all Brazilian regions ^(8)^.

The population estimates produced by IBGE are derived via demographic accounting methodology. This approach utilizes the most recent Population Census as a baseline and updates this figure annually by incorporating the components of population change: namely, births and deaths data from the civil registry system, and estimates of net migration, which are modeled using ancillary data sources such as school enrollments, health statistics, and prior demographic trends to project population counts for inter-censal periods ^(9)^.

To analyze utilization patterns, we calculated population-adjusted rates (total RAI prescriptions per 100,000 inhabitants) and procedure-adjusted rates, including the ratio of RAI prescriptions to oncologic thyroidectomies and the ratio of RAI prescriptions to estimated new DTC cases. Datasus oncologic thyroidectomy data are available from 2008 onward.

Temporal trends in RAI prescription patterns were assessed using segmented (joinpoint) regression analysis applied to log-transformed annual national prescription counts (2000-2024). This approach estimates each segment’s annual percent change (APC) and identifies statistically significant inflection points delineating distinct temporal trends. The optimal model was selected according to the Bayesian Information Criterion (BIC). Analyses were performed using R software (version 4.3.2, R Foundation for Statistical Computing, Vienna, Austria), and results were verified using the Joinpoint Trend Analysis Software (version 5.4.0, National Cancer Institute, Bethesda, MD, USA).

The study protocol received ethical approval from the Research Ethics Committee of the *Hospital de Clínicas de Porto Alegre* (CAAE 29670919.9.0000.5327/GPPG 2019-0764) and was conducted in accordance with ethical standards for observational research. The authors verified the accuracy of all data analyses and methodological approaches.

## RESULTS

### Number of DTC RAI prescriptions in Brazil

**Figure 1:**
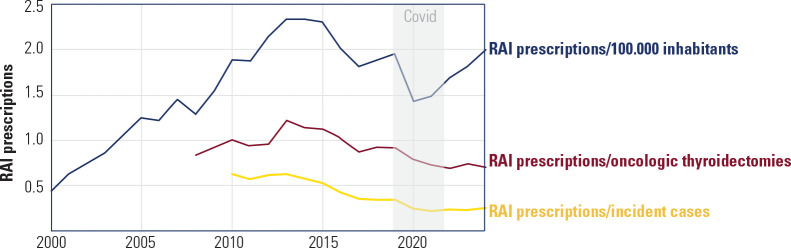
Population and thyroid cancer adjusted radioiodine (RAI) prescription trends for differentiated thyroid cancer (DTC), 2000-2024.

Analysis of RAI use for DTC in Brazil (2000-2024) revealed significant temporal trends in three key metrics: population-adjusted prescriptions, prescriptions per oncologic thyroidectomy, and prescriptions per new DTC case (**Figure 1**).

Data sources: DATASUS, INCA and IBGE.

Population-adjusted RAI prescriptions demonstrated a consistent upward trajectory from 2000 (0.44 per 100,000 inhabitants) to 2015 (2.30 per 100,000 inhabitants), followed by a gradual decline to 1.43 in 2020 during the COVID-19 pandemic, with subsequent recovery to 2.00 by 2024.

The most clinically informative metrics seems to be those adjusted for disease incidence: RAI prescriptions per oncologic thyroidectomy and per new DTC cases. The ratio of RAI prescriptions to oncologic thyroidectomies, available from 2008 onward, peaked at 1.22 in 2013 before decreasing to 0.70 in 2024 (42.6% reduction), indicating changing clinical practices in postoperative RAI administration. RAI prescriptions per new DTC cases showed a progressive decline from 0.63 in 2010 to 0.23-0.25 recently (2021-2024). As previously shown, the COVID-19 pandemic seems to have accelerated this trend, with the metric dropping sharply to 0.25 in 2020 and remaining at similarly reduced levels thereafter ^(10)^.

### RAI activities in Brazil

**Figure 2:**
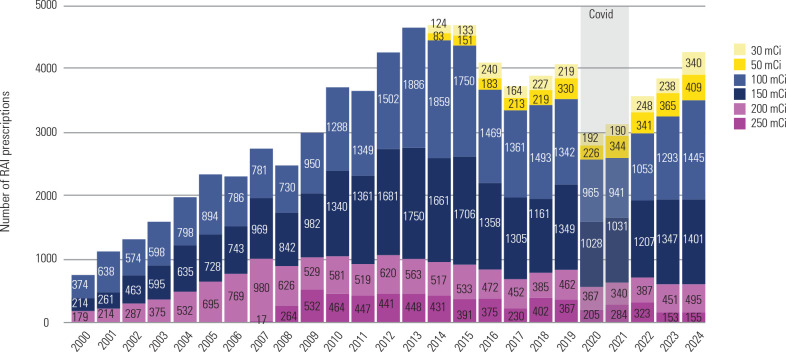
Radioiodine (RAI) activity prescriptions for differentiated thyroid cancer (DTC) in Brazil (2000-2024).

The analysis of RAI prescription data reveals significant evolution in treatment practices for DTC in Brazil over the 25-year study period. From 2000 to 2007, the early phase was characterized by exclusive use of activities ≥ 100 mCi, with total annual prescriptions increasing steadily from 767 to 2,747 (**Figure 2** and **Supplemental Table 1**). This period had a marked shift toward higher activities, with 200 mCi prescriptions growing 5.5-fold (from 179 to 980 prescriptions per year) and 150 mCi utilization increasing 4.5-fold (from 214 to 969 prescriptions per year).

Data source: DATASUS.

Between 2008-2015, RAI use peaked while showing early signs of change. Total prescriptions climbed to a maximum of 4,647 in 2013, with very high-activity prescriptions (≥ 200 mCi) accounting for 1,011 in the same year. Notably, 250 mCi use peaked in 2009 (532 prescriptions), while 100 mCi became the most frequently prescribed activity by 2013 (1,886 prescriptions). In 2014, the introduction of low-activity options (30/50 mCi) resulted in 207 total prescriptions, signaling a paradigm shift in therapeutic practice.

The most recent era (2016-2024) has been characterized by substantial de-escalation and diversification of RAI use. Very high-activity prescriptions (≥ 200 mCi) decreased by 35.7% from 2013 to 2024, while low-activity utilization demonstrated remarkable growth - 50 mCi prescriptions increased by 171% (from 151 in 2015 to 409 in 2024) and 30 mCi prescriptions rose by 156% (from 133 to 340) during the same period. The COVID-19 pandemic in 2020 temporarily disrupted this trend, causing a 28.3% single-year decline in total prescriptions, though post-pandemic recovery has maintained the trend toward lower RAI activities, which may be explained by the consolidation of guideline-based de-escalation of RAI and a still short post-pandemic observation period.

Segmented regression analysis objectively confirmed three distinct temporal phases in national RAI utilization. Two statistically significant joinpoints were identified in 2007 and 2015, corresponding to the onset of early diversification and subsequent de-escalation of RAI prescriptions. From 2000 to 2007, total RAI prescriptions increased markedly (APC = +17.2% per year, p < 0.01). Between 2008 and 2015, the rate of increase slowed but remained positive (APC = +4.1% per year, p = 0.02). From 2016 to 2024, a statistically significant decline was observed (APC = -3.9% per year, p < 0.01), consistent with the national adoption of selective, risk-adapted RAI practices. These findings quantitatively reinforce the descriptive three-phase narrative and demonstrate that the post-2015 reduction represents a true temporal inflection in Brazil’s RAI prescribing behavior.

Current prescription patterns in 2024 reflect a balanced utilization across activity levels, with very high-activities (200/250 mCi) accounting for 15.3% of prescriptions, high-activities (100/150 mCi) comprising 67.1%, and low-activities (30/50 mCi) making up 17.6% of total utilization (**Figure 2**). This evolution likely reflects the progressive adoption of risk-adjusted strategies, with more varied RAI activities and reduced dependence on very high/high-dose regimens. The rise of low-activity use since 2014 reflects Brazil’s alignment with international guidelines favoring more selective RAI application in DTC.

## DISCUSSION

Our nationwide analysis of RAI utilization patterns in Brazil from 2000 to 2024 reveals a significant transformation in DTC management, demonstrating the gradual adoption of up-to-date, evidence-based practices and persistent challenges in standardizing care. The trends reflect an important paradigm shift from routine to selective RAI use, though opportunities for further optimization remain.

Data from the United States and Europe illustrate a clear global trend toward de-escalating RAI for DTC, yet its adoption varies significantly by region ^(11,12)^. As observed in Brazil, the implementation of these protocols is often delayed, demonstrating that translating evidence-based guidelines into practice is highly dependent on local healthcare systems and context.

The most striking finding is the substantial decline in very high-activity RAI prescriptions (≥ 200 mCi), which decreased by 35.7% between 2013 and 2024, accompanied by a remarkable 163% average increase in low-activity (30-50 mCi) utilization during the same period. This evolution likely reflects the growing awareness of the thyroid cancer guidelines, reinforcing evidence of comparable outcomes with lower activities for low-risk DTC, while minimizing adverse effects and healthcare costs. This decline may also reflect the growing proportion of low-risk DTC cases recently, naturally reducing the indication for adjuvant RAI ^(13)^. The COVID-19 pandemic also led to an accelerated decline in RAI prescriptions, further reinforcing the viability of a more selective use of RAI therapy ^(10)^.

Our data reveal three distinct phases in Brazilian practice: (i) an early period (2000-2007) of rapidly increasing very high-activity utilization, (ii) a transitional phase (2008-2015) in which prescription patterns began diversifying, and (iii) the current era (2016-2024) characterized by progressive de-escalation. The introduction of low-activity options in 2014 marked a critical inflection point, with these regimens now comprising 17.6% of total prescriptions in 2024, which is a development that aligns with international trends toward individual patient risk-adapted therapy. These phases align closely with the emergence of evidence and guidelines advocating for more selective, risk adapted RAI therapy. The publication of 2009 American Thyroid Association (ATA) guidelines represented an initial paradigm shift, recommending against routine RAI for low-risk tumors ^(14)^. Subsequently randomized controlled trials in 2012, including the HiLo trial (UK) and the ESTIMABL1 trial (France), demonstrated non-inferiority of low-activity RAI (1.1 GBq or 30 mCi) compared to standard high-activity doses (3.7 GBq or 100 mCi) for remnant ablation in low-risk patients ^(15,16)^. These trials provided robust evidence supporting dose de-escalation. The 2015 ATA guidelines further reinforced this risk-stratified approach ^(2)^. The progressive adoption of these evidence-based recommendations in Brazil demonstrates successful translation of international clinical trial data and evolving consensus guidelines into local practice patterns.

Nevertheless, several challenges persist. The continued predominance of high-activities (67.1% of prescriptions) suggests potential over-treatment of low-risk patients, while the ongoing use of very high activities (15.3%) may reflect either appropriate management of high-risk cases or lingering adherence to outdated practices. These patterns likely stem from multiple factors, including regional variations in healthcare system structure and individual clinical practice ^(17)^.

The impact of reduced RAI use on oncologic outcomes remains a key consideration. Although this study cannot directly evaluate recurrence or mortality, long-term data from the HiLo and ESTIMABL1 trials suggest that low-dose RAI achieves similar ablation success and recurrence prevention compared to high-dose RAI in lowand intermediate-risk DTC, with no observed increase in recurrence or mortality ^(15,16)^. For high-risk DTC, however, RAI likely continues to provide a survival and recurrence benefit. A large multicenter prospective study of nearly 3,000 patients demonstrated improved overall survival and lower recurrence rates in high-risk patients receiving RAI ^(18)^, and national database analyses have shown that RAI remains protective for cancer-specific mortality primarily in high-risk subgroups ^(19)^.

The persistence of high-dose RAI (67.1% in 2024) may reflect past prescribing habits and heterogeneous adoption of guidelines across Brazilian regions. As shown in **Supplemental Table 2**, in the Brazilian Unified Health System (SUS), the remuneration for inpatient RAI therapy increases with administered activity, with distinct codes and higher payments for 100 to 250 mCi compared to lower doses (30 and 50 mCi), which are classified as ambulatory procedures with substantially lower reimbursement values. According to these data, for example, considering only the direct RAI costs, changing the prescription from 100 to 30 mCi represents a saving of R$ 628.20 per patient. Addressing these financial and behavioral drivers may assist a more uniform adoption of evidence-based, risk-adapted RAI therapy across Brazil. Notwithstanding, these economic factors were not assessed in this study.

The study’s findings have important policy implications for SUS. The reduction in RAI/new case ratio (from 0.63 to 0.25) suggests opportunities for significant cost savings via continued optimization of RAI use. While our findings are descriptive, they highlight the need for a national DTC registry to evaluate the long-term impact of these practice changes and to guide future RAI utilization policies and research in Brazil.

Our findings should be interpreted considering two main limitations: the use of administrative data and ecological design. These preclude analysis of individual-level clinical factors (e.g., tumor stage) and regional practice variations. Further studies are needed to correlate these utilization trends with patient outcomes and assess the economic consequences of practice pattern changes.

In conclusion, our findings demonstrate substantial progress in aligning Brazilian DTC management with international standards, while highlighting remaining opportunities for improvement. The documented evolution toward more selective RAI use reflects successful knowledge translation, though continued efforts are needed to ensure equitable adoption of evidence-based practices across all regions and healthcare settings. The results underscore the importance of ongoing monitoring and quality improvement initiatives to optimize thyroid cancer care in the Brazilian Unified Health System.

## Data Availability

the datasets used and/or analyzed during the current study available from the corresponding author on rea-sonable request.

## Supplementary MATERIALS

**Supplemental Table 1:** Number of radioiodine activities prescribed, and oncologic thyroidectomies performed in Brazil from 2000 to 2024

Year	Radioiodine therapy for Differentiated Thyroid Cancer						Oncologic Thyroidectomies
	30 mCi	50 mCi	100 mCi	150 mCi	200 mCi	250 mCi	All
2000	-	-	374	214	179	-	-
2001	-	-	638	261	214	-	-
2002	-	-	574	463	287	-	-
2003	-	-	598	595	375	-	-
2004	-	-	798	635	532	-	-
2005	-	-	894	728	695	-	-
2006	-	-	786	743	769	-	-
2007	-	-	781	969	980	17	-
2008	-	-	730	842	626	264	2,938
2009	-	-	950	982	529	532	3,268
2010	-	-	1,288	1,340	581	464	3,665
2011	-	-	1,349	1,361	519	447	3,920
2012	-	-	1,502	1,681	620	441	4,424
2013	-	-	1,886	1,750	563	448	3,814
2014	124	83	1,859	1,661	517	431	4,094
2015	133	151	1,750	1,706	533	391	4,153
2016	240	183	1,469	1,358	472	375	4,030
2017	164	213	1,361	1,305	452	230	4,260
2018	227	219	1,493	1,161	385	402	4,210
2019	219	330	1,342	1,349	462	367	4,439
2020	192	226	965	1,028	367	205	3,785
2021	190	344	941	1,031	340	284	4,287
2022	248	341	1,053	1,207	387	323	5,109
2023	238	365	1,293	1,347	451	153	5,210
2024	340	409	1,445	1,401	495	155	6,034

Data source: TABNET/DATASUS (tabnet.datasus.gov.br)

**Supplemental Table 2:** Unified Health System Thyroid Cancer Radioiodine Therapy Activities and Oncological Thyroidectomy Procedures Codes

Procedure	Datasus Code(s)	Period of Code Validity	Reimbursement Values (in Brazilian reais)
Radioiodine therapy for DTC 30 mCi	03.04.09.005-0	From Feb/2014 to present	R$ 443.70
Radioiodine therapy for DTC 50 mCi	03.04.09.006-9	From Feb/2014 to present	R$ 614.70
Radioiodine therapy for DTC 100 mCi	03.04.09.002-6	From Jan/2008 to present	R$ 1,071.90
	85.300.888 and 85.500.887	From Nov/1999 to Dec/2007	
Radioiodine therapy for DTC 150 mCi	03.04.09.001-8	From Jan/2008 to present	R$ 1,289.90
	85.300.900 and 85.500.909	From Nov/1999 to Dec/2007	
Radioiodine therapy for DTC 200 mCi	03.04.09.003-4	From Jan/2008 to present	R$ 1,471.32
	85.300.926 and 85.500.925	From Nov/1999 to Dec/2007	
Radioiodine therapy for DTC 250 mCi	03.04.09.004-2	From Jul/2008 to present	R$ 1,810.32
	03.03.12.001-0	From Jan/2008 to Jun/2008	
Total thyroidectomy in oncology	0416030270	From 2013 to present	R$ 2,836.30
	0416030130	From Aug/2008 to 2013	
Total thyroidectomy with lymph node resection	0416030122	From Jan/2008 to 2013	R$ 767.77
	0402010051	From Jan/2008 to present	
Transsternal thyroid tumor resection in oncology	0416030360	From 2013 to present	R$ 4,186.64
	0416030050	From Jan/2008 to 2013	

Data source: DATASUS.

Data source: TABNET/DATASUS (tabnet.datasus.gov.br).

DTC: differentiated thyroid cancer.

## References

[1] International Agency for Research on Cancer Global Cancer Observatory.

[2] Haugen BR, Alexander EK, Bible KC, Doherty GM, Mandel SJ, Nikiforov YE (2016). 2015 American Thyroid Association Management Guidelines for Adult
Patients with Thyroid Nodules and Differentiated Thyroid
Cancer. Thyroid.

[3] Mallick U, Newbold K, Beasley M, Garcez K, Wadsley J, Johnson SJ (2025). Thyroidectomy with or without postoperative radioiodine for
patients with low-risk differentiated thyroid cancer in the UK (IoN): a
randomised, multicentre, non-inferiority trial. Lancet.

[4] Leboulleux S, Bournaud C, Chougnet CN, Lamartina L, Zerdoud S, Do Cao C (2024). Thyroidectomy without radioiodine in patients with low-risk
thyroid cancer: 5 years of follow-up of the prospective randomised ESTIMABL2
trial. Lancet Diabetes Endocrinol.

[5] Ringel MD, Sosa JA, Baloch Z, Bischoff L, Bloom G, Brent GA (2025). 2025 American Thyroid Association Management Guidelines for Adult
Patients with Differentiated Thyroid Cancer. Thyroid.

[6] Schwengber WK, Mota LM, Nava CF, Rodrigues JAP, Zanella AB, Kuchenbecker RS (2021). Patterns of radioiodine use for differentiated thyroid carcinoma
in Brazil: insights and a call for action from a 20-year
database. Arch Endocrinol Metab.

[7] Shrime MG, Goldstein DP, Seaberg RM, Sawka AM, Rotstein L, Freeman JL (2007). Cost-effective management of low-risk papillary thyroid
carcinoma. Arch Otolaryngol Head Neck Surg.

[8] Instituto Nacional de Câncer (INCA) (2023). Estimativa 2023: Incidência de Câncer no Brasil.

[9] Instituto Brasileiro de Geografia e Estatística
(IBGE) (2024). Metodologia da Estimativa da População dos
Municípios para 1º de Julho de 2024.

[10] Silveira VB, Schwengber WK, Hetzel GM, Zanella AB, Scheffel RS, Maia AL (2022). Effect of COVID-19 pandemic on diagnosis and treatment of thyroid
cancer in Brazil. Front Endocrinol (Lausanne).

[11] Dal Maso L, Pierannunzio D, Francisci S, De Paoli A, Foffolutti F, Vaccarella S (2023). Trends in radioactive iodine treatment after total thyroidectomy
in Italy, 2001-2018. Eur Thyroid J.

[12] Pasqual E, Sosa JA, Chen Y, Schonfeld SJ, González AB, Kitahara CM. (2022). Trends in the Management of Localized Papillary Thyroid Carcinoma
in the United States (2000-2018). Thyroid.

[13] Chen DW, Haymart MR. (2025). Unravelling the rise in thyroid cancer incidence and addressing
overdiagnosis. Nat Rev Endocrinol.

[14] Cooper DS, Doherty GM, Haugen BR, Kloos RT, Lee SL, Mandel SJ (2009). Revised American Thyroid Association management guidelines for
patients with thyroid nodules and differentiated thyroid
cancer. Thyroid.

[15] Schlumberger M, Leboulleux S, Catargi B, Deandreis D, Zerdoud S, Bardet S (2018). Outcome after ablation in patients with low-risk thyroid cancer
(ESTIMABL1): 5-year follow-up results of a randomised, phase 3, equivalence
trial. Lancet Diabetes Endocrinol.

[16] Dehbi HM, Mallick U, Wadsley J, Newbold K, Harmer C, Hackshaw A (2019). Recurrence after low-dose radioiodine ablation and recombinant
human thyroid-stimulating hormone for differentiated thyroid cancer (HiLo):
long-term results of an open-label, non-inferiority randomised controlled
trial. Lancet Diabetes Endocrinol.

[17] Padovani RP, Pansani IF, Marone MMS, Vaisman F, Maia AL, Dora JM (2024). Physicians’ preferences for radioiodine treatment of
differentiated thyroid cancer in Brazil: an observational
study. Arch Endocrinol Metab.

[18] Jonklaas J, Sarlis NJ, Litofsky D, Ain KB, Bigos ST, Brierley JD (2006). Outcomes of patients with differentiated thyroid carcinoma
following initial therapy. Thyroid.

[19] Ruel E, Thomas S, Dinan M, Perkins JM, Roman SA, Sosa JA. (2020). Adjuvant radioactive iodine therapy is associated with improved
survival for patients with intermediate-risk papillary thyroid
cancer. Endocr Relat Cancer.

